# Impact of nutritional indices on mortality in patients with heart failure

**DOI:** 10.1136/openhrt-2017-000730

**Published:** 2018-01-09

**Authors:** Akiomi Yoshihisa, Yuki Kanno, Shunsuke Watanabe, Tetsuro Yokokawa, Satoshi Abe, Makiko Miyata, Takamasa Sato, Satoshi Suzuki, Masayoshi Oikawa, Atsushi Kobayashi, Takayoshi Yamaki, Hiroyuki Kunii, Kazuhiko Nakazato, Hitoshi Suzuki, Takafumi Ishida, Yasuchika Takeishi

**Affiliations:** Department of Cardiovascular Medicine, Fukushima Kenritsu Ika Daigaku, Fukushima, Japan

**Keywords:** heart failure, nutrition, malnutrition, metabolism, exercise capacity, prognosis

## Abstract

**Background:**

Malnutrition is a common condition that is associated with adverse prognosis in patients with heart failure (HF). The Prognostic Nutritional Index (PNI), Geriatric Nutritional Risk Index (GNRI) and controlling nutritional status (CONUT) have all been used as objective indices for evaluating nutritional status. We aimed to clarify the relationship between these nutritional indices and the parameters of inflammatory markers, cardiac function and exercise capacity, as well as to compare the ability of these indexes for predicting mortality.

**Methods:**

We evaluated PNI, GNRI and CONUT in consecutive 1307 patients with HF.

**Results:**

First, there were significant correlations between nutritional indices and the following: C reactive protein; tumour necrosis factor-α; adiponectin; B-type natriuretic peptide; troponin I; inferior vena cava diameter and peak VO_2_ (P<0.05, respectively). Second, in the Kaplan-Meier analysis (follow-up 1146 days), all-cause mortality progressively increased from normal to mild, moderate and severe disturbance groups in the indices (log-rank, P<0.01, respectively). In the Cox proportional hazard analysis, each index was an independent predictor of all-cause mortality in patients with HF (P<0.001, respectively). Third, receiver operating curve demonstrated that the areas under the curve of PNI and GNRI were larger than that of CONUT score (P<0.05, respectively).

**Conclusion:**

Patients with HF being malnourished had higher mortality accompanied by higher levels of C reactive protein, tumour necrosis factor-α, adiponectin, B-type natriuretic peptide, troponin I, right-sided volume overload and impaired exercise capacity, rather than left ventricular systolic function. Additionally, PNI and GNRI were superior to CONUT score in predicting mortality in patients with HF.

**Key questions**

**What is already known about this subject?**

Several nutritional indices (eg, Prognostic Nutritional Index (PNI), Geriatric Nutritional Risk Index (GNRI) and controlling nutritional status (CONUT)) have been reported to be useful to assess in patients with heart failure (HF). However, the relationship between these nutritional indices and other factors (eg, inflammatory markers, renal function, haemoglobin, cardiac function and exercise capacity) remains unclear. In addition, it remains unclear which index is more useful for estimating prognosis in patients with HF.

**What does this study add?**

Over 20% of hospitalised patients with HF have moderate-to-severe malnutrition determined by PNI, GNRI and CONUT score. Patients with HF being malnourished had higher mortality accompanied by higher levels of B-type natriuretic peptide, troponin I, C reactive protein, tumour necrosis factor-α, adiponectin, right-sided volume and pressure overload and impaired exercise capacity, but not with systolic function of the left and right ventricles. In addition, PNI and GNRI were superior to CONUT score in predicting mortality in patients with HF.

**How might this impact on clinical practice?**

Nutritional indices is useful to predict prognosis in patients with HF.

## Introduction

Heart failure (HF) is a major cause of death among the elderly in many countries and has become a significant public health problem.^1 2^ HF causes low nutritional intake due to intestinal oedema and anorexia, absorption disorder and increases resting metabolic rate, large energy and nutrient demand of human heart^3^ and leads to malnutrition. In turn, malnutrition leads to exacerbation of fluid retention, inflammation, neurohormonal activation^4^ and as a result is associated with adverse prognosis in patients with HF.^3^ Although body mass index (BMI) is a well-known assessment tool of nutritional status and is associated with prognosis in patients with HF,^5^ it does not necessarily indicate precise nutritional status due to fluid retention caused by HF.

Appropriate nutritional assessment is important, and several nutritional indices have been reported to assess patients with chronic disease or general population. The Prognostic Nutritional Index (PNI),^6^ the Geriatric Nutritional Risk Index (GNRI)^7^ and controlling nutritional status (CONUT)^8^ require simple objective markers (ie, serum albumin levels, serum total cholesterol levels, total lymphocyte count and body weight), and are widely used for evaluating nutritional status in elderly patients or patients with chronic disease. The PNI can be used to predict the mortality of patients with liver disease, as well as the quality of life of these patients.^6^ The Nutritional Risk Index (NRI) is a nutrition-related risk index that makes it possible to classify patients according to a risk of morbidity and mortality in relation to pathologies,^9^ and the GNRI is a revised version of the NRI, specifically adapted for the elderly patients.^7^ A CONUT score is an efficient tool for early detection and continuous control of undernutrition in hospital, and allows assessment of nutritional status in all patients.^8^

Several studies have reported the utility of risk stratification of these indices for predicting prognosis in patients with HF.^4 10–14^ However, the relationship between these nutritional indices and other known important prognostic factors (eg, inflammatory markers, renal function, haemoglobin, cardiac function and exercise capacity) remains unclear. In addition, it remains unclear which index is more useful for estimating prognosis in patients with HF.

Thus, we aimed to clarify the relationship between nutritional indices and parameters of inflammatory markers, cardiac function and exercise capacity, as well as to compare the ability of these indices to predict mortality.

## Methods

### Subjects and study protocol

**Figure 1:**
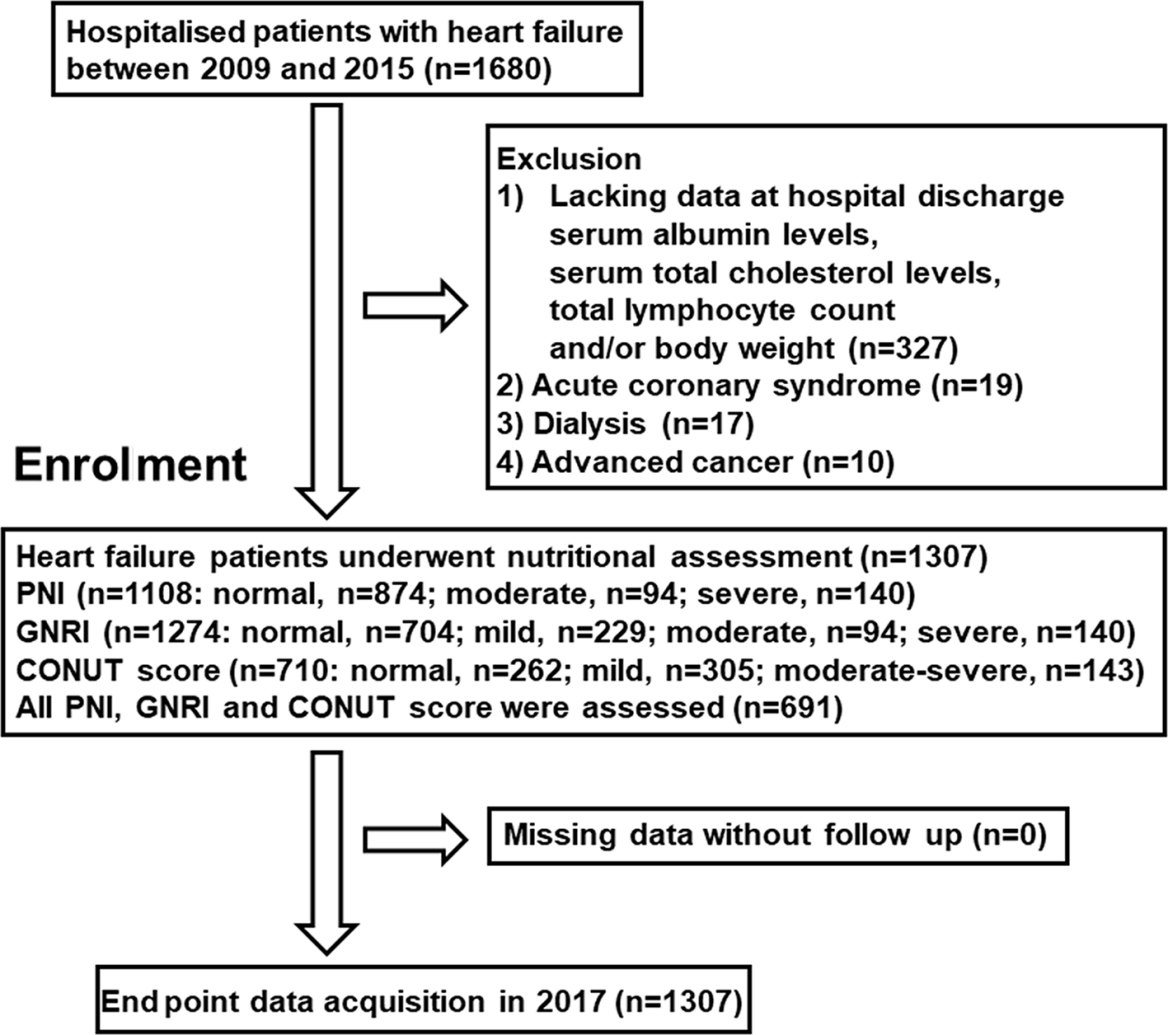
Patient flow chart. CONUT, controlling nutritional status; GNRI, Geriatric Nutritional Risk Index; PNI, Prognostic Nutritional Index.

This was a retrospective study that enrolled consecutive 1680 patients with symptomatic HF, who had been hospitalised for treatment of decompensated HF, and discharged from Fukushima Medical University between January 2009 and December 2015. The diagnosis of decompensated HF was made by several cardiologists based on the HF guidelines.^1 2^ Blood samples were obtained at discharge. We calculated PNI, GNRI and CONUT score as previously reported.^6–8^ Patients lacking data of one or more of the components of these scores (ie, serum albumin, serum total cholesterol, total lymphocyte count and body weight), those who had acute coronary syndrome, those who received dialysis and those who had distinct advanced cancer were excluded. The patients’ flow is shown in figure 1.

We performed several examinations, such as general laboratory tests, echocardiography and cardiopulmonary exercise tests at discharge, and compared each parameter with each nutritional index. Comorbidities were also assessed by several attending physicians. Hypertension was defined as the recent use of antihypertensive drugs, a systolic blood pressure of ≥140 mm Hg and/or a diastolic blood pressure of ≥90 mm Hg. Diabetes was defined as the recent use of insulin or antidiabetic drugs, a fasting blood glucose value of > 126 mg/dL and/or a haemoglobin A1c value of > 6.5%. Dyslipidemia was defined as the recent use of cholesterol-lowering drugs, a triglyceride value of ≥150 mg/dL, a low-density lipoprotein cholesterol value of ≥140 mg/dL and/or a high-density lipoprotein cholesterol value of <40 mg/dL. Chronic kidney disease was defined as an estimated glomerular filtration rate (eGFR) of <60 mL/min/1.73 cm^2^. Anaemia was defined as haemoglobin levels of <12.0 g/dL in females and <13.0 g/dL in males.^1^ Atrial fibrillation was identified by an ECG performed during hospitalisation and/or from medical records including patients having a history of atrial fibrillation.

The patients were followed up until 2017 for all-cause death. The status and/or dates of death of all patients were obtained from the patients’ medical records or attending physicians at the patient’s referring hospital. We were able to follow-up all patients. Survival time was calculated from the date of hospitalisation until the date of death or last follow-up. Written informed consent was obtained from all study subjects at discharge. The study protocol was carried out in accordance with the principles outlined in the Declaration of Helsinki. Reporting of the study conforms to Strengthening the Reporting of Observational Studies in Epidemiology (STROBE) along with references to STROBE and the broader Enhancing the QUAlity and Transparency Of Health Research guidelines.^15^

### Nutritional assessment and classifications

The PNI was calculated as follows: PNI=10× serum albumin (g/dL)+0.005×total lymphocyte (count per mm^3^).^6^ Patients with a PNI >38 are considered as normal, those with a PNI of 35–38 are at moderate risk of malnutrition and those with a PNI <35 are at severe risk.^6^ The GNRI was calculated as follows: GNRI=14.89× serum albumin (g/dL)+41.7×body weight/ideal body weight.^7^ Ideal body weight=22× square of height in metres. Patients with a GNRI ≥98 are considered as normal, those with a GNRI of 92–97 are mild risk of malnutrition, those with a GNRI of 82–91 are at moderate risk and those with a GNRI <82 are at severe risk.^7^ The CONUT score was calculated by serum albumin and total cholesterol levels, and total lymphocyte counts as previously reported.^8^ Patients with a CONUT score of 0–1 have a normal nutritional status, those with a CONUT score of 2–4 are at mild risk of malnutrition, those with a CONUT score of 5–8 are at moderate risk and those with a CONUT score of 9–12 are at severe risk.^8^ These data were assessed at hospital discharge. Patients with severe nutritional status in CONUT score were only seven patients and gathered up moderate and severe CONUT status in this study.

### Echocardiography

Echocardiography was performed blindly by experienced echocardiographers using the standard techniques. The echocardiographic parameters included: left ventricular ejection fraction (LVEF); right ventricular fractional area change (RV-FAC); inferior vena cava diameter and tricuspid regurgitation pressure gradient (TRPG). The LVEF was calculated using Simpson’s method. The RV-FAC, defined as (end diastolic area−end systolic area)/end diastolic area×100, was a measure of right ventricular systolic function.^16^ All measurements were performed using ultrasound systems (ACUSON Sequoia, Siemens Medical Solutions USA, Mountain View, California, USA).

### Cardiopulmonary exercise testing

The patients underwent incremental symptom-limited exercise testing using an upright cycle ergometer with a ramp protocol before discharge (Strength Ergo 8, Fukuda Denshi, Tokyo, Japan). Breath-by-breath oxygen consumption (VO_2_), carbon dioxide production (VCO_2_) and minute ventilation (VE) were measured during exercise using an AE-300S respiratory monitor (Minato Medical Science, Osaka, Japan). Peak VO_2_ was measured as an average of the last 30 s of exercise. Ventilatory response to exercise (slope of the relationship between ventilation and carbon dioxide production, VE/VCO_2_ slope) was calculated as the regression slope relating VE to CO_2_ from the start of exercise until the respiratory compensation point (the time at which ventilation is stimulated by CO_2_ output and end-tidal CO_2_ tension begins to decrease).^17^ The ventilatory anaerobic threshold was calculated using the V-slope method.

### Measurement of levels of TNF-α, adiponectin and troponin I

A blood sample was obtained from each patient at Fukushima Medical University before discharge under a fasting state. The plasma B-type natriuretic peptide (BNP) levels were measured using a specific immunoradiometric assay (Shionoria BNP kit, Shionogi, Osaka, Japan). The troponin I was measured in EDTA anticoagulated plasma using a refined assay (Abbott-Architect; Abbott Laboratories, Abbott Park, Illinois, USA). Tumour necrosis-factor (TNF)-α was measured, based on the method of solid phase chemiluminescent ELISA using an immunoassay kit (Quanti Glo ELISA Human TNF-α Immunoassay, R&D Systems, Minneapolis, USA). Total serum adiponectin was based on the method of latex agglutination turbidimetric immunoassay, also using an immunoassay kit (Human Adiponectin Latex Immunoassay, LSI Medience, Tokyo, Japan).

### Statistical analysis

Normally distributed data are presented as mean±SD, and non-normally distributed data were log transformed (ie, C reactive protein, BNP, troponin I, TNF-α, adiponectin). The categorical variables are expressed as numbers and percentages, and the χ^2^ test was used for comparisons. We used analysis of variance followed by Bonferroni’s post hoc test. Correlations between each nutritional score and data of demographics, laboratory test, echocardiography and cardiopulmonary exercise test were assessed using Spearman’s correlation analysis. The Kaplan-Meier method was used for presenting mortality, with the log-rank test. The prognostic value was tested by univariate and multivariate Cox proportional hazard analyses. The proportional hazards assumption for the model was checked by examining log minus-log transformed. The Kaplan-Meier estimates of the survival curves for three groups were plotted against the time to follow-up period. These curves help in identifying non-proportionality patterns in hazard function such as convergent (difference in risk among the groups decreases with time), divergent or crossing of the curves. In addition, the Schoenfeld test for violation of proportional hazards, which assesses the correlation between scaled residuals and time, was also conducted. To assess the each nutritional index and each component of the scores to predict mortality, we also estimated the areas under the curves (AUC) of the receiver operating curve (ROC) to compare with other known biomarkers using the DeLong test. P<0.05 was considered statistically significant for all comparisons. These analyses were performed using a statistical software package (SPSS V.24.0, IBM, Armonk, New York, USA).

## Results

Over 20% of hospitalised patients with HF have moderate-to-severe nutritional disturbance determined by PNI, GNRI and CONUT score (21.1% in PNI; 26.8% in GNRI and 20.1% in CONUT score: figure 1). The demographic, laboratory test, echocardiographic and cardiopulmonary exercise data of the present study’s subjects (mean age 66.5 years, male 792, mean LVEF 42.2%) in the nutritional indices (PNI, GNRI and CONUT score) are summarised in online supplementary tables 1–3. Low PNI and GNRI, and a high CONUT score indicate nutritional disorder. In summary, depending on the degree of each nutritional disorder, age, heart rate, New York Heart Association (NYHA) class, C reactive protein, BNP, troponin I, TNF-α, adiponectin, inferior vena cava diameter, TRPG tended to increase, whereas BMI, haemoglobin, total lymphocyte count, total protein, albumin, sodium, eGFR, total cholesterol and peak VO_2_ tended to decrease. In contrast, LVEF and RV-FAC did not differ among the groups. Additionally, correlation analyses with the nutritional indices and other parameters are presented in table 1. There were many significant correlations between each nutritional indices and age, BMI, heart rate, NYHA class, haemoglobin, total protein, sodium, eGFR, total cholesterol, C reactive protein, BNP, troponin I, TNF-α, adiponectin, inferior vena cava diameter, TRPG and peak VO_2_ (P<0.05, respectively), but no correlations were observed between any of the indices and either LVEF or RV-FAC. In addition, there were significant correlations between the nutritional indices and the other nutritional indices.

10.1136/openhrt-2017-000730.supp1 Supplementary file 1

**Table 1:** Correlation analyses with nutritional indices and other parameters

	PNI (n=1108)	GNRI (n=1274)	CONUT (n=710)
Demographic data			
Age	−0.263**	−0.253**	0.219**
Body mass index	0.099**	0.685**	−0.122**
Systolic blood pressure	−0.044	0.021	0.067
Diastolic blood pressure	0.011	0.037	0.038
Heart rate	−0.176**	−0.182**	0.194**
NYHA class	−0.211**	−0.219**	0.205**
Laboratory data			
Haemoglobin	0.516**	0.493**	−0.533**
Total lymphocyte count	0.699**	0.207**	−0.438**
Total protein	0.567**	0.501**	−0.513**
Albumin	0.849**	0.785**	−0.794**
Sodium	0.269**	0.265**	−0.272**
eGFR	0.257**	0.214**	−0.264**
Total cholesterol	0.309**	0.287**	−0.545**
Log C reactive protein	−0.529**	−0.399**	0.535**
Log BNP	−0.392**	−0.443**	0.378**
Log troponin I	−0.267**	−0.219**	0.233**
Log TNF-α	−0.168*	−0.150*	0.273*
Log adiponectin	−0.305**	−0.390**	0.475**
Echocardiography			
LVEF	0.049	0.097	−0.088
RV-FAC	0.027	0.001	−0.016
IVC	−0.110**	−0.009	0.142**
TRPG	−0.074*	−0.076*	0.114*
Cardiopulmonary exercise test			
Peak VO_2_	0.205**	0.176**	−0.189**
VE/VCO_2_ slope	−0.143**	−0.232**	0.100
Nutritional indices			
PNI	–	0.684**	−0.809**
GNRI	0.684**	–	−0.651**
CONUT	−0.809**	−0.651**	–

*P<0.05, **P<0.01.

BNP, B-type natriuretic pepide; CONUT, controlling nutritional status; eGFR, estimated glomerular filtration; GNRI, Geriatric Nutritional Risk Index; IVC, inferior vena cava diameter; LVEF, left ventricular ejection fraction; NYHA, New York Heart Association; peak VO_2_, breath-by-breath oxygen consumption; PNI, Prognostic Nutritional Index; RV-FAC, right ventricular fractional area change; TNF-α, tumour necrosis factor-α; TRPG, tricuspid valve regurgitation; VE/VCO_2_ slope, slope of the relationship between ventilation and carbon dioxide production.

**Figure 2:**
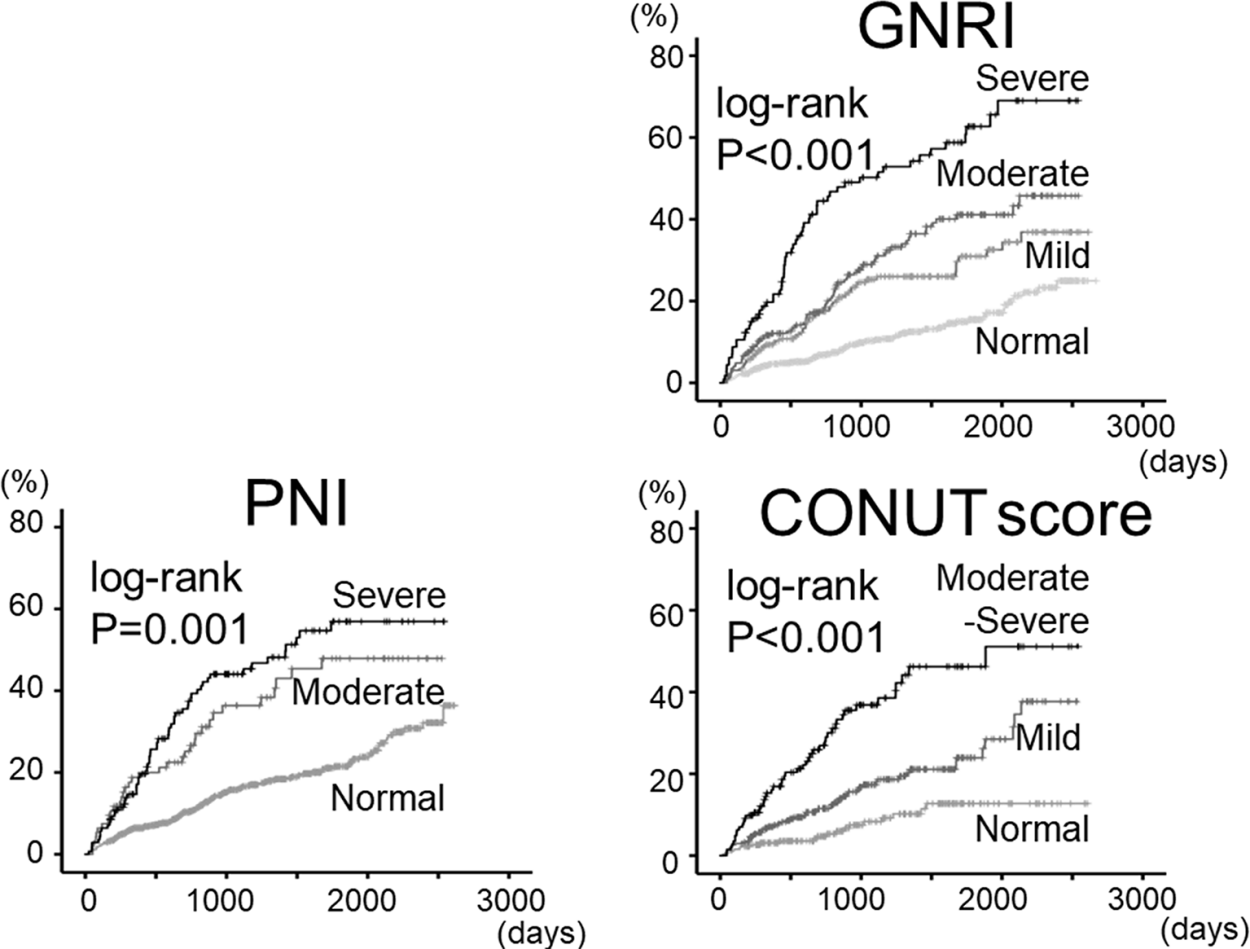
Cumulative all-cause mortality stratified by PNI, GNRI and COUNT scores. Kaplan-Meier analysis for all-cause mortality in the indices. CONUT, controlling nutritional status; GNRI, Geriatric Nutritional Risk Index; PNI, Prognostic Nutritional Index.

**Figure 3:**
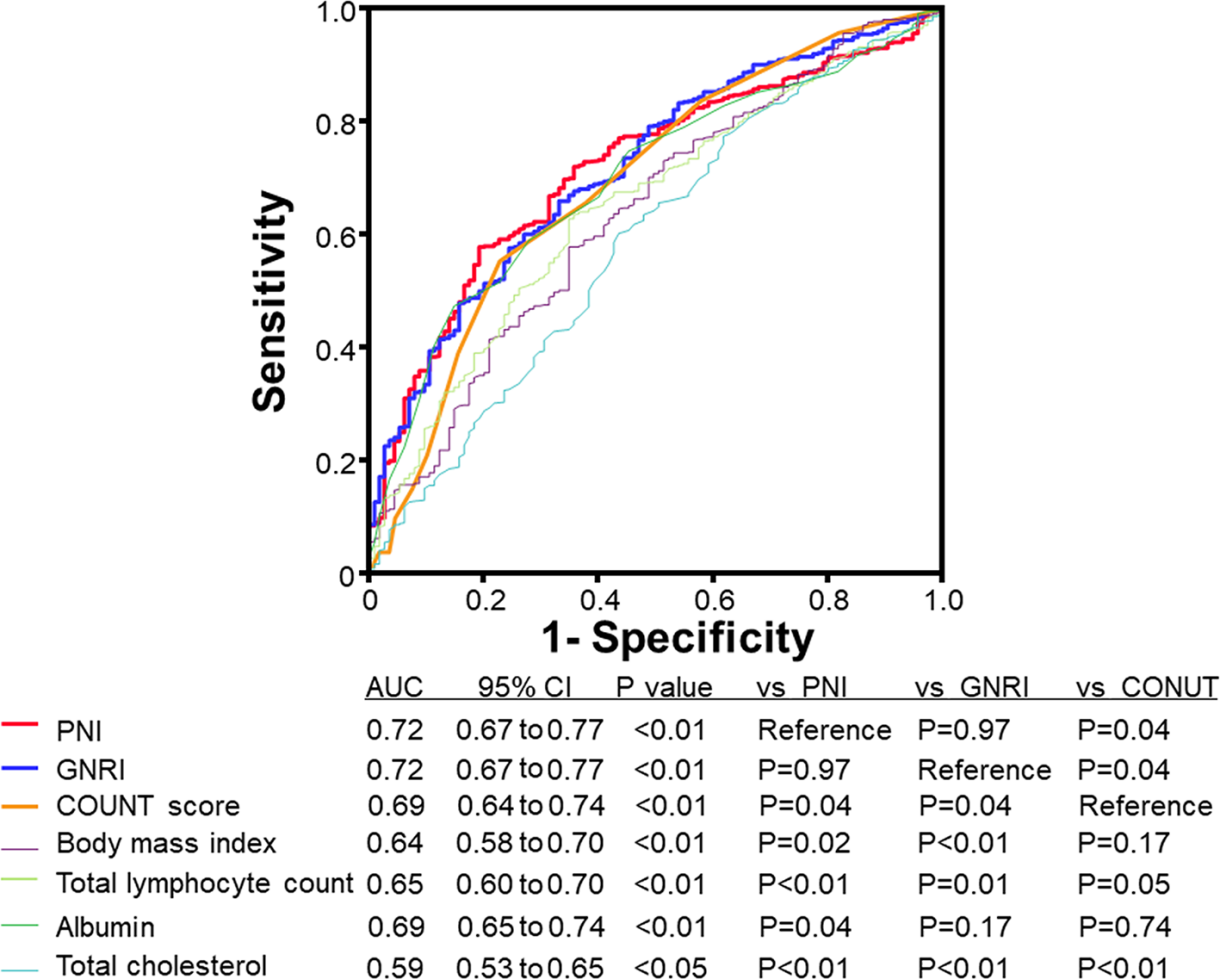
Receiver operating curve (ROC) to predict mortality in patients with HF (n=691). AUC, area under the curve; CONUT, controlling nutritional status; GNRI, Geriatric Nutritional Risk Index; PNI, Prognostic Nutritional Index.

Regarding prognosis (mean follow–up 1146 days), as shown in figure 2, all-cause mortality progressively increased from normal to mild, moderate and severe nutritional disturbance groups in the nutritional indices (P<0.01, respectively). After adjusting for other potential confounding factors, the Cox proportional hazard analysis (table 2) revealed that PNI, GNRI and CONUT score were independent predictors of all-cause mortality in patients with HF (P<0.01, respectively). In addition, the ROC curve (figure 3) demonstrated that the AUCs of PNI and GNRI were significantly higher than that of CONUT score, and AUC of PNI, GNRI and CONUT score were larger than those of the components of these scores (ie, albumin, total cholesterol, total lymphocyte count and BMI) in patients with HF (n=691).

**Table 2:** Univariable and multivariable Cox proportional hazard model of all-cause mortality

	HR	95% CI	P value
PNI (one category increase) (event=248/n=1108)			
Unadjusted	1.484	1.350 to 1.630	<0.001
Adjusted model 1*	1.319	1.192 to 1.460	<0.001
Adjusted model 2†	1.178	1.051 to 1.321	0.005
GNRI (one category increase) (event=281/n=1274)			
Unadjusted	1.751	1.580 to 1.940	<0.001
Adjusted model 1*	1.548	1.385 to 1.731	<0.001
Adjusted model 2†	1.372	1.210 to 1.556	<0.001
CONUT (one category increase) (event=125/n=710)			
Unadjusted	1.795	1.480 to 2.178	<0.001
Adjusted model 1*	1.445	1.163 to 1.796	0.001
Adjusted model 2†	1.387	1.100 to 1.749	0.006

*Adjusted for age, sex, systolic blood pressure, heart rate and New York Heart Association class.

†Adjusted for model 1+presence of ischaemic aetiology, hypertension, diabetes, dyslipidemia, chronic kidney disease, anaemia, atrial fibrillation, B-type natriuretic peptide and left ventricular ejection fraction.

CONUT, controlling nutritional status; GNRI, Geriatric Nutritional Risk Index; PNI, Prognostic Nutritional Index.

## Discussion

In the present study, over 20% of the hospitalised patients with HF were determined to have moderate-to-severe malnutrition according to their PNI, GNRI and/or CONUT score. To the best of our knowledge, the current study is the first to show that patients with HF being malnourished had higher mortality accompanied by higher levels of BNP, troponin I, C reactive protein, TNF-α, adiponectin, right-sided volume and pressure overload, and impaired exercise capacity, but not with systolic function of the left and right ventricles. This study is also the first to compare PIN, GNRI and CONUT score in same cohort, as well as to emphasise that PNI and GNRI were superior to CONUT score in predicting mortality in patients with HF.

The utility of nutritional status as a predictor of mortality in patients with HF has been reported. It has been recently reported that lower PNI is associated with mortality in patients with acute decompensated HF with reduced or preserved LVEF.^10^ Lower NRI is associated with higher readmission rate and mortality in patients with acute decompensated HF,^12^ and mortality in outpatients with chronic HF.^13^ Lower GNRI was significantly associated with mortality in hospitalised patients with HF with preserved LVEF,^14^ as well as those with acute decompensated HF.^18^ Higher CONUT score is associated with increased rate of HF hospitalisation and mortality in the elderly with cardiovascular disease.^19^ Another report suggested that PNI, GNRI and CONUT score were independently associated with cardiovascular events, and that CONUT score was relatively superior for predicting outcome compared with PNI and GNRI.^11^ Contrary to this report,^11^ our present data showed that PNI and GNRI were superior to CONUT score in predicting mortality in patients with HF. Out of our 1680 patients with HF, 76.3% (n=1282) had dyslipidemia, and the usage of statins in the present study was 39.4% (n=662) and was higher than that of previous report as of 25%.^11^ Thus, frequencies of dyslipidemia and the statin use affect total cholesterol level, a component of CONUT score, and might influence predictive value of CONUT. Therefore, PNI and GNRI seem to be more suitable than CONUT for predicting mortality in patients with HF taking statins.

Another nutritional test, the Mini Nutritional Assessment (MSA), was developed with the primary goal of assessing nutritional status in the elderly,^20^ and is considered to be the strongest predictor of mortality in HF.^4^ However, MSA required the assessment of many factors, including general (residential status, psychological problems, mobility, medication and skin ulcers), anthropometric factors (BMI, arm and calf circumferences and weight loss), dietary factors (meals, food and fluid intake and autonomy of feeding) and subjective data (self-perception of health and nutrition). Thus, it is difficult to evaluate MSA in all patients in general. PNI, GNRI and CONUT score are all relatively simpler than MSA, and are easily applicable for patients with HF. The components of each index (ie, albumin, total cholesterol, total lymphocyte count, BMI) have been reported as risk factors in patients with HF. In the current study, we have presented that: 1) the predictive values of the nutritional indices were superior compared with those of albumin, total cholesterol, total lymphocyte count and BMI; 2) there were significant correlations between PNI, GNRI and CONUT score and 3) PNI and GNRI were superior to CONUT score in predicting mortality in patients with HF.

Associations between nutritional indices (ie, PNI, GNRI and CONUT) and other important factors are assumed. Malnutrition is associated with increased right-sided volume overload (eg, inferior vena cava diameter), rather than LV systolic dysfunction.^21–23^ Cardiac cachexia is related to haemodynamic alterations of HF,^22^ and the following neurohumoral responses have in turn been implicated in gastrointestinal function,^21^ liver function, anorexia, negative energy balance, systemic inflammation/catabolism (eg, C reactive protein, TNF-α and adiponectin), decreased lean, fat mass and skeletal muscle and leads to impaired exercise capacity (eg, peak VO_2_) in patients with HF.^3 24^ Additionally, these neurohumoral responses and systemic inflammation is associated with myocardial damage and cardiac overload (eg, troponin I and BNP).^25^ Concordant with these previous findings, there were significant correlations between C reactive protein, TNF-α, adiponectin, BNP, troponin I, inferior vena cava diameter, peak VO_2_ and nutritional indices in the present study.

Regarding the relationships between the nutritional indices and other prognostic factors are assumed. Total lymphocyte count, which is a component of PNI, is positively associated with haemoglobin and inversely associated with age, central venous pressure, creatinine, leucocyte count and soluble TNF receptor-1.^26^ It has been recently reported that PNI is correlated with haemoglobin, eGFR, sodium and right ventricular systolic pressure,^10^ as well as current study. The NRI, which is the origin of the GNRI, is related to metabolic and inflammatory biomarkers, and appetite-regulatory hormones (eg, ghrelin, adiponectin, TNF-α, pentraxin-3 and natriuretic peptide),^12^ and is not associated with left ventricular function.^13^ The GNRI is associated with upper and lower extremity muscle mass, handgrip strength, knee extensor muscle strength, inspiratory and expiratory muscle pressure and exercise capacity (eg, VE/VCO_2_ slope) in patients with chronic HF.^27^ In addition, there are significant correlations between nutritional scores (MSA, GNRI and CONUT) and both C reactive protein and natriuretic peptides in patients with decompensated HF.^28^ Concordant with these findings, our data revealed that the group with more nutritional disturbance had higher mortality accompanied by higher levels of BNP, troponin I, C reactive protein, TNF-α, adiponectin, right-sided volume and pressure overload (ie, inferior vena cava diameter and TRPG). The same group had lower levels of haemoglobin and peak VO_2_, and these are reportedly prognostic factors of patients with HF.^1 2^

### Study strengths and limitations

Our study has some strengths. First, we performed comparisons between nutritional indices for predicting mortality, with considering other known important prognostic factors comprehensively (eg, cardiac function, renal function, haemoglobin, inflammatory markers and exercise capacity). Second, our study population was relatively larger than those of previous studies,^11 13 14 18^ and we were able to follow-up all patients.

There are several limitations in the present study. First, we did not consider any changes in any parameters, and baseline data at admission were used for the analyses. Second, although we conducted multivariable analyses to evaluate associations between the nutritional indices and prognosis in patients with HF, confounding factors could be entirely eliminated. Further studies with a larger population are needed.

## Conclusions

Over 20% of hospitalised patients with HF have moderate-to-severe malnutrition determined by PNI, GNRI and CONUT score. In the current study, patients with HF being malnourished had higher mortality accompanied by higher levels of BNP, troponin I, C reactive protein, TNF-α, adiponectin, right-sided volume and pressure overload and impaired exercise capacity. PNI and GNRI were superior to CONUT score in predicting mortality in patients with HF.
