# Supplementary material for: Impact of nutritional indices on mortality in patients with heart failure
Source: Open Heart. 2018 Jan 9;5(1):e000730. doi: 10.1136/openhrt-2017-000730 (PMC5761292; doi:10.1136/openhrt-2017-000730)
Supplement: Supplementary file 1 [file openhrt-2017-000730supp001.pdf]

Supplementary Table 1. Comparisons of clinical features among PNI category (N=1108)

	Normal (*n*=874)	Moderate (*n*=94)	Severe (*n*=140)	*P*-value
PNI score	47.7 ± 6.5	36.8 ± 3.9**	30.5 ± 3.8**††	<0.001
Age (years)	65.3 ± 14.2	70.8 ±15.8**	72.3 ± 14.7**	<0.001
Male gender (*n*, %)	552 (63.2)	50 (53.2)	66 (47.1)	0.001
Body mass index (kg/cm^2)	23.5 ± 4.0	23.4 ± 4.7	22.9 ± 4.9	0.300
Systolic blood pressure (mmHg)	126.9 ± 28.8	129.1 ± 31.4	130.4 ± 33.7	0.371
Diastolic blood pressure (mmHg)	72.2 ± 19.2	72.8 ± 19.2	71.4 ± 21.3	0.851
Heart rate (bpm)	78.1 ± 22.8	85.0 ± 24.1*	91.6 ± 27.0**	<0.001
NYHA class III or IV (*n*, %)	20 (2.3)	2 (2.1)	11 (7.9)	0.004
Ischemic etiology (*n*, %)	222 (25.4)	24 (25.5)	40 (28.6)	0.800
**Co-morbidity**				
Hypertension (*n*, %)	626 (71.6)	66 (70.2)	98 (70.0)	0.005
Diabetes (*n*, %)	321 (36.7)	46 (48.9)	65 (46.4)	0.025
Dyslipidemia (*n*, %)	681 (77.9)	68 (72.3)	117 (83.6)	0.206
Chronic kidney disease (*n*, %)	439 (50.2)	66 (70.2)	93 (66.4)	<0.001
Anemia (*n*, %)	386 (44.2)	67 (71.3)	119 (85.0)	<0.001
Atrial fibrillation (*n*, %)	320 (36.6)	30 (31.9)	55 (39.3)	0.702
**Laboratory data**				
Hemoglobin (g/dl)	13.1 ± 2.1	11.6 ± 2.2**	10.3 ± 2.3**††	<0.001
Total lymphocyte count (mm^3)	1633.3 ± 968.1	1074.2 ± 433.0**	884.2 ± 389.7**	<0.001
Total protein (g/dl)	7.1 ± 0.6	6.5 ± 0.8**	6.0 ± 0.9**††	<0.001
Albumin (g/dl)	4.0 ± 0.4	3.1 ± 0.2**	2.6 ± 0.4**††	<0.001
Sodium (mEq/l)	139.4 ± 3.2	137.4 ± 4.8**	136.8 ± 5.1**	<0.001
eGFR (ml/min/1.73 cm^2)	58.6 ± 22.9	44.7 ± 30.4**	46.6 ± 23.9**	<0.001
Total cholesterol (mg/dl)	181.5 ± 37.9	163.5 ± 38.0**	148.0 ± 47.7**	<0.001
Log C-reactive protein (mg/dl)	-0.77 ± 0.66	-0.12 ± 0.76**	0.28 ± 0.69**††	<0.001
Log BNP (pg/ml)	2.3 ± 0.6	2.8 ± 0.6**	2.7 ± 0.5**	<0.001
Log troponin I (ng/ml)	-1.2 ± 0.8	-0.8 ± 0.9**	-0.6 ± 0.9**††	<0.001
Log TNF-α (pg/ml)	0.3 ± 0.4	0.3 ± 0.4	0.4 ± 0.4	0.712
Log adiponectin (μg/ml)	1.2 ± 0.3	1.4 ± 0.4	1.4 ± 0.2*	0.009
**Echocardiography**				
LVEF (%)	42.7 ± 16.0	42.2 ± 14.9	42.4 ± 15.6	0.945
RV-FAC (%)	40.1 ± 12.3	42.9 ± 12.3	40.0 ± 11.2	0.408
IVC (mm)	14.6 ± 4.6	16.2 ± 4.8	15.2 ± 5.6	0.074
TRPG (mmHg)	29.9 ± 16.8	32.8 ± 15.8	31.4 ± 14.4	0.386
**Cardiopulmonary exercise test**				

Peak VO_2 (ml/kg/min)	16.0 ± 4.8	15.2 ± 4.5	13.5 ± 3.2*	0.029
VE/VCO_2 slope	34.2 ± 8.2	35.8 ± 9.0	36.8 ± 8.2	0.241

NYHA, New York Heart Association; eGFR, estimated glomerular filtration; BNP, B-type natriuretic peptide; TNF-α, tumor necrosis factor-α; LVEF, left ventricular ejection fraction; RV-FAC, right ventricular fractional area change; IVC, inferior vena cava diameter; TRPG, tricuspid valve regurgitation; Peak VO_2, breath-by-breath oxygen consumption; VE/VCO_2 slope, slope of the relationship between ventilation and carbon dioxide production. *$P<0.05$ and **$P<0.01$ vs. normal group, †$P<0.05$ and ††$P<0.01$ vs. moderate group.

Supplementary Table 2. Comparisons of clinical features among GNRI category (N=1274)

	Normal (*n*=704)	Mild (*n*=229)	Moderate (*n*=227)	Severe (*n*=114)	*P*-value
GNRI score	107.9 ± 7.5	95.0 ± 1.7**	87.6 ± 2.7**	75.5 ± 5.5**††‡‡	<0.001
Age (years)	63.6 ± 14.2	68.7 ± 13.0**	69.9 ± 14.6**	70.9 ± 16.1**	<0.001
Male gender (*n*, %)	456 (64.8)	133 (58.1)	137 (60.4)	53 (46.5)	0.002
Body mass index (kg/cm^2)	25.3 ± 4.0	21.8 ± 2.7**	21.3 ± 2.9**	19.0 ± 2.9**††‡‡	<0.001
Systolic blood pressure (mmHg)	126.9 ± 26.8	127.8 ± 31.7	128.6 ± 32.4	124.6 ± 36.1	0.680
Diastolic blood pressure (mmHg)	72.4 ± 18.2	74.3 ± 20.8	71.0 ± 20.2	71.8 ± 21.6	0.315
Heart rate (bpm)	76.6 ± 20.9	80.4 ± 25.1	84.4 ± 25.2**	91.3 ± 27.7**††	<0.001
NYHA class III or IV (*n*, %)	10 (1.4)	6 (2.6)	7 (3.1)	12 (10.5)	<0.001
Ischemic etiology (*n*, %)	183 (26.0)	55 (24.0)	67 (29.5)	26 (22.8)	0.470
**Co-morbidity**					
Hypertension (*n*, %)	528 (75.0)	159 (69.4)	172 (75.8)	73 (64.0)	0.037
Diabetes (*n*, %)	270 (38.4)	86 (37.6)	100 (44.1)	41 (36.0)	0.361
Dyslipidemia (*n*, %)	565 (80.3)	173 (75.5)	169 (74.4)	89 (78.1)	0.207
Chronic kidney disease (*n*, %)	351 (49.9)	122 (53.3)	142 (62.6)	72 (63.2)	0.001
Anemia (*n*, %)	269 (38.2)	127 (55.5)	172 (75.8)	92 (80.7)	<0.001
Atrial fibrillation (*n*, %)	256 (36.4)	90 (39.3)	80 (35.2)	41 (36.0)	0.817
**Laboratory data**					
Hemoglobin (g/dl)	13.5 ± 2.0	12.5 ± 2.0**	11.5 ± 2.2**††	10.3 ± 2.2**††‡‡	<0.001
Total lymphocyte count (mm^3)	1599.3 ± 952.0††	1456.7 ± 902.0††	1274.2 ± 688.4**	1057.0 ± 546.8**	<0.001
Total protein (g/dl)	7.3 ± 0.6	6.9 ± 0.6 **	6.6 ± 0.8**††	6.1 ± 1.0 **††‡‡	<0.001
Albumin (g/dl)	4.1 ± 0.4	3.6 ± 0.3 **	3.2 ± 0.4 **††	2.7 ± 0.5 **††‡‡	<0.001
Sodium (mEq/l)	139.8 ± 3.0	138.8 ± 3.4**	137.8 ± 4.3**†	137.1 ± 5.3**††	<0.001
eGFR (ml/min/1.73 cm^2)	60.6 ± 23.1	55.3 ± 23.4	49.4 ± 25.6**	47.1 ± 25.7**	<0.001
Total cholesterol (mg/dl)	184.8 ± 39.6	178.6 ± 37.8	166.1 ± 36.0**	141.2 ± 46.4**††‡‡	<0.001
Log C-reactive protein (mg/dl)	0.84 ± 0.61	0.58 ± 0.74**	-0.26 ± 0.75**††	0.10 ± 0.76**††‡‡	<0.001
Log BNP (pg/ml)	2.2 ± 0.6	2.4 ± 0.5**	2.7 ± 0.5**††	2.8 ± 0.5**††	<0.001
Log troponin I (ng/ml)	-1.2 ± 0.7	-1.0 ± 0.8*	-0.9 ± 0.8**	-0.6 ± 0.9**††‡‡	<0.001
Log TNF-α (pg/ml)	0.3 ± 0.4	0.3 ± 0.5	0.4 ± 0.5	0.4 ± 0.4	0.066
Log adiponectin (μg/ml)	1.1 ± 0.3	1.3 ± 0.3**	1.4 ± 0.2**	1.4 ± 0.3**	<0.001

**Echocardiography**					
LVEF (%)	43.1 ± 15.9	40.1 ± 16.8	41.4 ± 14.7	41.0 ± 16.5	0.082
RV-FAC (%)	40.8 ± 12.1	41.1 ± 11.5	38.9 ± 13.7	40.6 ± 10.9	0.570
IVC (mm)	14.6 ± 4.5	15.3 ± 5.6	15.1 ± 5.1	14.4 ± 4.8	0.264
TRPG (mmHg)	29.0 ± 15.4	30.2 ± 16.8	32.7 ± 18.5	31.4 ± 13.8	0.089
**Cardiopulmonary exercise test**					
Peak VO_2 (ml/kg/min)	16.4 ± 4.6	15.1 ± 5.3	14.8 ± 4.5	13.9 ± 3.0	0.004
VE/VCO_2 slope	32.9 ± 7.4	36.2 ± 8.4**	37.0 ± 9.5**	34.9 ± 6.6	<0.001

NYHA, New York Heart Association; eGFR, estimated glomerular filtration; BNP, B-type natriuretic peptide; TNF-α, tumor necrosis factor-α; LVEF, left ventricular ejection fraction; RV-FAC, right ventricular fractional area change; IVC, inferior vena cava diameter; TRPG, tricuspid valve regurgitation; Peak VO_2, breath-by-breath oxygen consumption; VE/VCO_2 slope, slope of the relationship between ventilation and carbon dioxide production. *P<0.05 and **P<0.01 vs. normal group, †P<0.05 and ††P<0.01 vs. mild group, ‡P<0.05 and ‡‡P<0.01 vs. moderate group.

Supplementary Table 3. Comparisons of clinical features among CONUT score category (N=710)

	**Normal (*n*=262)**	**Mild (*n*=305)**	**Moderate-Severe (*n*=143)**	***P*-value**
CONUT score	0.6±0.5	2.8±0.8**	6.7±1.9**††	<0.001
Age (years)	62.2±13.5	67.1±13.4**	71.0 ± 14.8**††	<0.001
Male gender (*n*, %)	169 (64.5)	204 (66.9)	76 (53.1)	0.017
Body mass index (kg/cm^2)	24.1±4.1	23.1 ± 3.8*	22.8 ± 5.0**	0.003
Systolic blood pressure (mmHg)	123.0 ± 22.7	122.5 ± 26.7	130.4 ± 32.3*††	0.008
Diastolic blood pressure (mmHg)	69.6 ± 14.8	69.8 ± 18.1	71.9 ± 19.1	0.403
Heart rate (bpm)	74.6 ± 19.6	75.0 ± 19.0	86.2 ± 24.4**††	<0.001
NYHA class III or IV (*n*, %)	3 (1.1)	8 (2.6)	8 (5.6)	0.030
Ischemic etiology (*n*, %)	51 (19.5)	78 (25.6)	35 (24.5)	0.207
**Co-morbidity**				
Hypertension (*n*, %)	164 (62.6)	209 (68.5)	96 (67.1)	0.316
Diabetes (*n*, %)	82 (31.3)	119 (39.0)	71 (49.7)	0.001
Dyslipidemia (*n*, %)	212 (80.9)	233 (76.4)	109 (76.2)	0.364
Chronic kidney disease (*n*, %)	111 (42.4)	166 (54.4)	91 (63.6)	<0.001
Anemia (*n*, %)	65 (24.8)	151 (49.5)	107 (74.8)	<0.001
Atrial fibrillation (*n*, %)	93 (35.5)	118 (38.7)	55 (38.5)	0.709
**Laboratory data**				
Hemoglobin (g/dl)	14.0 ± 1.6	12.7 ± 1.9 **	11.0 ± 2.3**††	<0.001
Total lymphocyte count (mm^3)	2061.5 ± 1211.1	1229.1 ± 451.6**	931.3 ± 375.2**††	<0.001
Total protein (g/dl)	7.3 ± 0.6	7.0 ± 0.6 **	6.3 ± 0.9 **††	<0.001
Albumin (g/dl)	4.2 ± 0.4	3.9 ± 0.4 **	2.9 ± 0.5 **††	<0.001
Sodium (mEq/l)	140.4 ± 2.5	139.1 ± 3.5	137.4 ± 5.1**††	<0.001
eGFR (ml/min/1.73 cm^2)	63.2 ± 19.9	54.7 ± 23.1**	47.8 ± 24.9**†	<0.001
Total cholesterol (mg/dl)	198.8 ± 33.9	172.9 ± 35.0**	143.3 ± 38.0**††	<0.001
Log C-reactive protein (mg/dl)	-0.97 ± 0.49	-0.74 ± 0.67**	0.001 ± 0.71**††	<0.001
Log BNP (pg/ml)	2.1 ± 0.5	2.3 ± 0.6**	2.8 ± 0.5**††	<0.001
Log troponin I (ng/ml)	-1.4 ± 0.5	-1.3 ± 0.7*	-1.0 ± 0.8**††	<0.001
Log TNF-α (pg/ml)	0.2 ± 0.2	0.2 ± 0.2	0.4 ± 0.4*†	0.011
Log adiponectin (μg/ml)	1.0 ± 0.3	1.1 ± 0.3	1.4 ± 0.2**††	<0.001
**Echocardiography**				
LVEF (%)	44.6 ± 16.2	42.2 ± 16.0	41.1 ± 15.7	0.077
RV-FAC (%)	40.9 ± 11.4	40.2 ± 12.5	41.5 ± 14.2	0.763
IVC (mm)	13.9 ± 3.8	14.6 ± 4.5	16.0 ± 6.1	<0.001
TRPG (mmHg)	29.5 ± 18.4	30.0 ± 16.0	34.8 ± 17.8	0.038
**Cardiopulmonary exercise test**				

Peak VO_2 (ml/kg/min)	16.2 ± 4.3	15.4 ± 4.4	14.7 ± 4.0	0.195
VE/VCO_2 slope	34.4 ± 7.7	34.5 ± 9.2	36.2 ± 8.1	0.557

NYHA, New York Heart Association; eGFR, estimated glomerular filtration; BNP, B-type natriuretic peptide; TNF-α, tumor necrosis factor-α; LVEF, left ventricular ejection fraction; RV-FAC, right ventricular fractional area change; IVC, inferior vena cava diameter; TRPG, tricuspid valve regurgitation; Peak VO_2, breath-by-breath oxygen consumption; VE/VCO_2 slope, slope of the relationship between ventilation and carbon dioxide production.
*P<0.05 and **P<0.01 vs. normal group, †P<0.05 and ††P<0.01 vs. mild group.
